# Complex motivations of Japanese medical students to an online medical English course during the COVID-19 pandemic

**DOI:** 10.12688/mep.19042.1

**Published:** 2022-04-06

**Authors:** Miu Azuma, Osamu Nomura, Takaya Sakuma, Yuki Soma

**Affiliations:** 1 School of Medicine, Hirosaki University, Hirosaki, Aomori, 036-8562, Japan; 2 Department of Emergency and Disaster Medicine, Hirosaki University, Hirosaki, Aomori, 036-8562, Japan; 3 Center for Postgraduate Education and Training, National Center for Child Health and Development, Setagaya-ku, Tokyo, 157-8535, Japan; 4 Faculty of Education, Hirosaki University, Hirosaki, Aomori, 036-8560, Japan

**Keywords:** Medical English, medical students, motivation, predictors, performance

## Abstract

**Background:** In response to globalism, many East-Asian countries now include a Medical English course in their undergraduate medical education syllabus. The purpose of this study was to explore the relationship between the related attribute factors of students' motivation to learn medical English through an online modality.

**Methods:** Of 134 eligible fourth-year medical students who participated in an Online Medical English course at a Japanese medical school, 105 were enrolled in this single cohort study. The participants completed pre- and post-course surveys regarding their motivation during the course, including perceived academic control and task value, and their assignment scores. A structural equation model was used to examine the hypothesized relationship of constructs, based on control-value theory.

**Results:** The model showed a good fit for the data (χ ^2^[df=7] = 1.821, p=0.969, CFI = 1.000, RMSEA < 0.001, SRMR < 0.05, GFI = 0.993, AGFI = 0.980). The latent variables of the perceived course achievement related to the observed variables of academic control and task value scale scores, and negatively predicted willingness for self-study after course completion. In addition, the preference of English as the course language negatively predicted willingness for self-study of medical English.

**Conclusion:** Choice of English as the language of instruction and perceived high course achievement negatively predicted students’ motivation for further English self-study after the class. The importance of incorporating the perspective of lifelong learning into the teaching of medical English was recognized.

## Introduction

Globalization has become a strong force affecting healthcare and education in the health professions worldwide ^1^. Health professions education is now an international enterprise due to the globalization of healthcare delivery, with increasing collaboration between medical schools and hospitals in different countries. Increasing movement of individuals between countries for business and tourism purposes now requires health professionals even in non-English speaking countries to communicate with foreign patients in English ^2, 3^. In some non-English-speaking countries, English has emerged as the medium of instruction for teaching medical education subjects ^4^. Furthermore, the global response to the COVID-19 pandemic has highlighted the use of English as the essential "international language" by health professionals and biomedical researchers, and the use of English has enabled prompt dissemination and gathering of medical research findings that have saved patients’ lives ^5^.

Accordingly, a medical English course is now included in the curriculum of health professions education in East-Asian countries, including Japan, Korea, and China ^6– 10^. However, traditional educational culture is a potential barrier for teaching and learning medical English in these countries. In East Asia, Confucianism is the fundamental value in the teaching and learning community, and the educational culture reflects the Confucian ideals of filial piety, loyalty to state, submission to authority, and social order; thus, the traditional modality of teaching is a didactic lecture in which students are expected to listen quietly ^11^. Although didactic lectures can effectively provide students with medical knowledge, this teaching method is less effective in learning English for communication purposes and has the effect of decreasing students' motivation. Furthermore, large-scale teaching makes it difficult for instructors to give students feedback and to create classroom interactions between students and instructors.

Online technology-based learning has been recognized as an effective instructional strategy for teaching medical English in the East Asian medical education context as it is a rich modality of communication that enhances student-centeredness ^8^. Students can initiate learning modules at a convenient time and place, and watch videos of lectures recorded by the instructors, which promotes student-centered and self-regulated learning. The students submit their assignments via an online platform according to the deadline, and instructors can grade the assignments and provide individualized feedback even when the students number more than 100. This system can foster teacher–learner interactions and facilitate student motivation, and it is also a powerful solution for issues associated with educational delivery in the COVID-19 pandemic era ^12^. The student–teacher interaction can be maintained via the online platform even when the participants are socially and physically distant.

To maximize the effectiveness of online medical education courses, it is vital to explore attribute factors of the course such as students’ motivation and the instructor’s feedback to the students. By identifying the attributes of successful medical English courses, the program faculty can improve the course structure and promote students' self-regulated learning of medical English. Therefore, this study aimed to explore the relationship of related attributive factors of students’ motivation to learn medical English via an online modality.

## Methods

### Educational context

This study was conducted at Hirosaki University in Japan. An Online Medical English course was provided for fourth-year medical students from July to August in 2020. The course consisted of three modules: (1) cultural diversity, (2) sending emails in English, and (3) delivering an elevator pitch. In each module, the students were required to watch a 30-minute instructional video, complete the assigned task for each topic, and submit the assignment. Classes were delivered in the Japanese language based on the students’ responses to a pre-class survey regarding the preferred course language.

### Study design and participants

**Figure 1.**
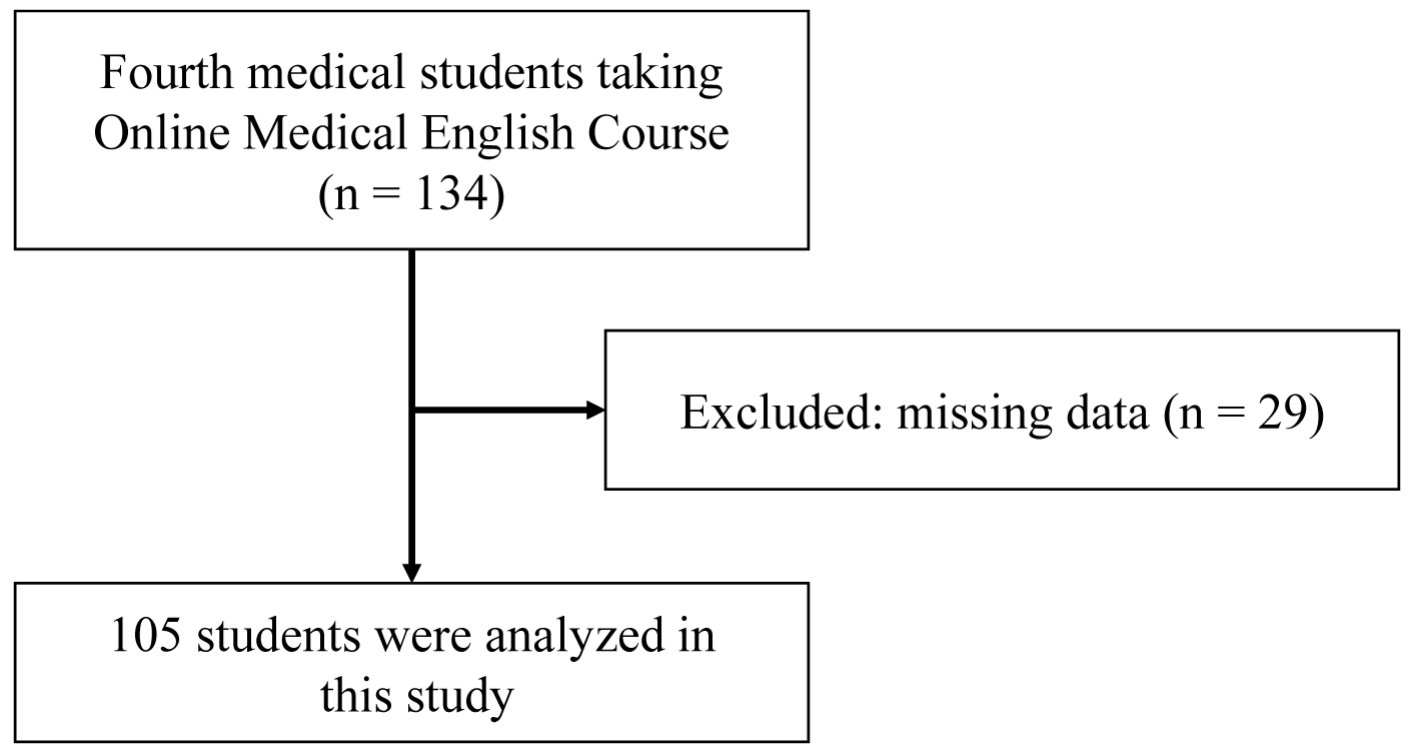
Flow chart of participant selection.

The Online Medical Education database of our university was utilized in this single cohort study. Of the 134 eligible fourth-year medical students at our medical school, 29 were excluded due to missing data, and 105 students were enrolled ( Figure 1).

### Data collection

The database included pre- and post-course surveys regarding motivation on the course, as well as the assignment scores. We first conducted a pre-course survey that asked whether the students preferred the classes to be delivered in English or Japanese on the day of the first module in July 2020. The assignments for the first two modules were graded by the instructor based on a predefined grading rubric. The post-survey, conducted on the day of the last module in August 2020, included items for measurement of perceived academic control and task value, and asked whether the students wanted to undertake further self-study in medical English using extra-curricular materials. Academic control was assessed using the five items in the Japanese version of the Academic Control Scale to measure their cognitive appraisal of control-toward-performance ^13, 14^. The perceived value of the task was assessed using the six items in the Japanese version of the Motivated Strategies for Learning Questionnaire ^15^. A copy of the pre- and post-course surveys can be found in the Extended data.

### Theoretical framework

**Figure 2.**
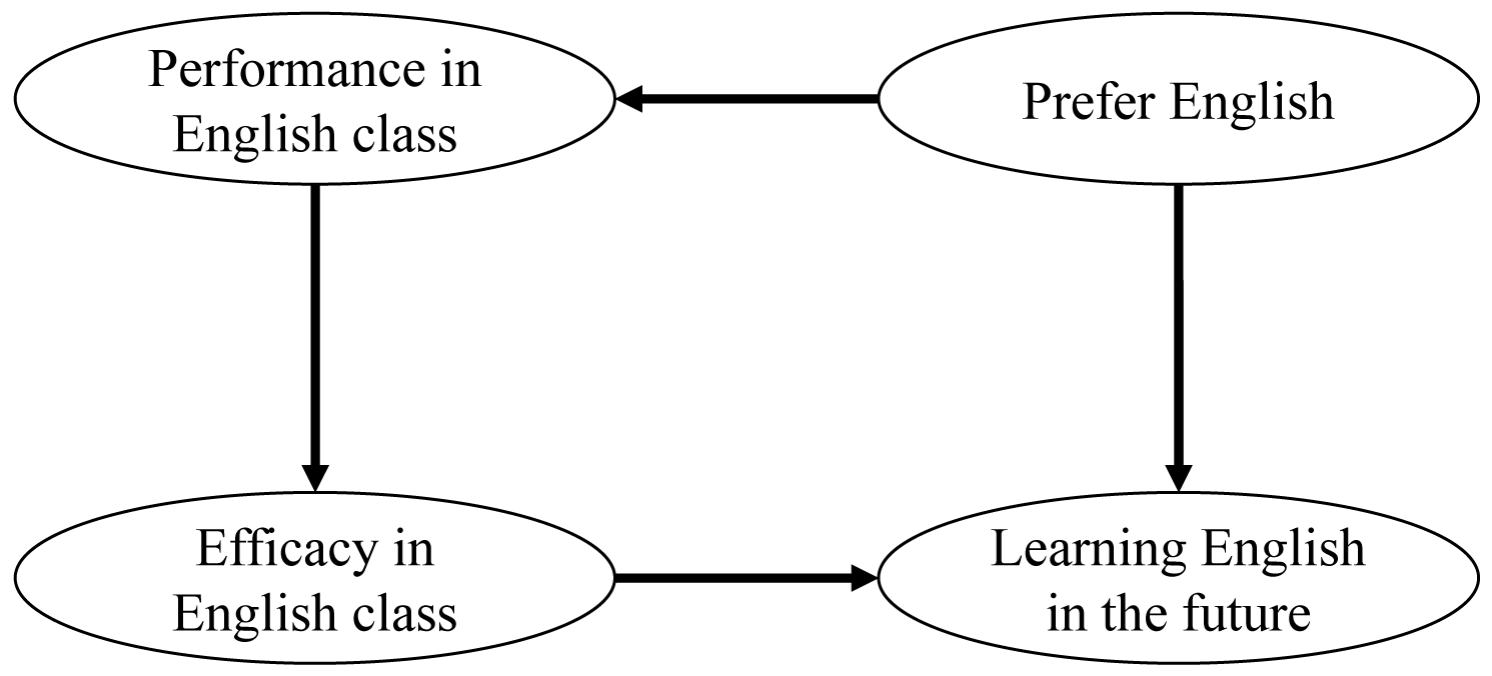
Hypothesis of the relationship of the constructs. A circulatory relationship among the variables of language delivery in the course, performance in class, efficacy in the English class, and willingness to undertake self-study after completion of the course.

We applied Pekrun’s control-value theory as the theoretical framework for the analysis. This theory postulates that the learners’ perceptions of control and value are predictors of the learners’ academic achievement ^16, 17^. Accordingly, we hypothesized a circulatory relationship among the variables of language delivery in the course, performance in class, efficacy in the English class, and willingness to undertake self-study after completion of the course. In other words, that students who prefer English as the course language score higher in the assignment, their score performance predicts higher scores of academic control and task value in the post-course survey (class efficacy), and class efficacy promotes students’ motivation to undertake self-study after completion of the class ( Figure 2).

### Statistical analysis

**Figure 3.**
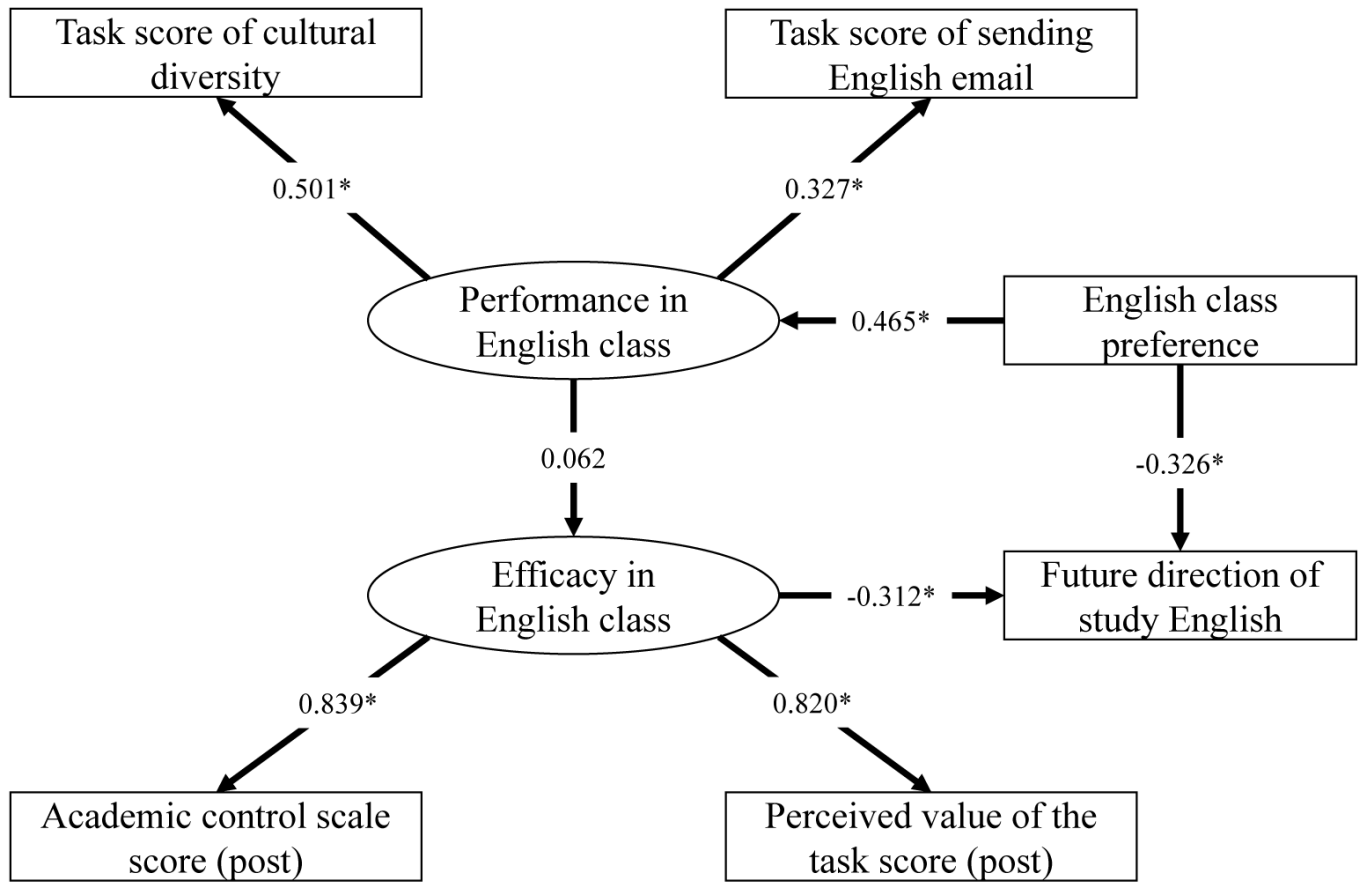
Structural model results. Fit indices: *χ* ^2^ = 1.821 (df = 7, p = 0.969), CFI = 1.000, RMSEA < 0.001, SRMR < 0.05, GFI = 0.993, AGFI = 0.980, *p-value < 0.05.

We conducted univariate statistical analyses from two aspects of comparison: (1) preferred course language ( Table 2) and (2) willingness towards self-study of medical English after course completion ( Table 3). Wilcoxon rank sum test or chi-square test was performed for the analyses, as appropriate. A structural equation model was used to examine the hypothesized relationship of the constructs, based on control-value theory ( Figure 3). The goodness of fit of the model was determined by examining the following: comparative fit index (CFI), root-mean-square error of approximation (RMSEA), standardized root-mean-squared residual (SRMR), goodness of fit index (GFI), and adjusted goodness of fit index (AGFI). All statistical analyses were conducted using the R software (version 4.1.0), and lavaan (version 0.6-8), semPlot (version 1.1.2), and semTools (version 0.5-4) packages.

### Ethics

The written informed consent of the study participants was waived, and the informed consent was obtained on an opt-out basis. This consent process was reviewed by the Ethics Committee of the Hirosaki University Graduate School of Medicine. The Ethics Committee of the university waived the ethics approval process as this study is not an interventional study involving patients but an educational observational study whose participants were medical students (Decision date: January 8 ^th^, 2021).

## Results

### Univariate analysis

Table 1 summarizes the descriptive statistics of the measured variables. Students who preferred English as the course language scored higher in the cultural diversity assignment. There was no significant difference in score in the assignment on sending English emails, or in scores for academic control or task value, in terms of choice of course language ( Table 2). The percentage of students willing to study medical English after completion of the course was significantly lower in students who preferred English as the course language than in those who preferred Japanese ( Table 3).

**Table 1.** Characteristics of participants.

	n = 105
Preferred English as the course language	41 (39.0)
Score of cultural diversity assignment	17 (15–20)
Score of sending English emails assignment	32 (30–36)
Academic control scale score	4.0 (3.6–4.2)
Perceived value of the task score	5.5 (4.9–6.0)
Willingness for self-study of medical English	42 (40.0)

Note. Values are the median (25th–75th percentile) or n (percentage).

**Table 2.** Comparison of variables according to preference of course language.

	Preferred English n = 41	Preferred Japanese n = 64	p-value
Score of cultural diversity assignment	20 (16–20)	16 (15–19)	0.004
Score of sending English emails assignment	32 (32–36)	32 (30–32.5)	0.154
Academic control scale score	4.0 (3.4–4.2)	4.0 (3.6–4.2)	0.589
Task value scale score	5.5 (5.0–6.0)	5.5 (4.8–6.0)	0.461
Willingness for self-study of medical English	8 (19.5)	34 (53.1)	< 0.001

Note. Values are the median (25th–75th percentile) or n (percentage). Analysis was performed by Wilcoxon rank sum test or chi-square test, as appropriate.

**Table 3.** Comparison of variables according to willingness to study English after the course.

	Willing n = 42	Unwilling n = 63	p-value
Preferred English as the course language	8 (19.0)	33 (52.4)	< 0.001
Score of cultural diversity assignment	16 (15–20)	17 (15–20)	0.414
Score of sending English emails assignment	32 (30–32)	32 (32–36)	0.146
Academic control scale score	3.8 (3.3–4.0)	4.0 (3.8–4.2)	0.004
Perceived value of the task score	5.2 (4.6–5.7)	5.6 (5.0–6.1)	0.003

Note. Values are the median (25th–75th percentile) or n (percentage). Analysis was performed by Wilcoxon rank sum test or chi-square test, as appropriate.

### Structural equation modeling

The model showed a good fit for the data (χ ^2^[df=7] = 1.821, p=0.969; CFI = 1.000, RMSEA < 0.001, SRMR < 0.05, GFI = 0.993, AGFI = 0.980). The latent variable of course performance was related to the observed variables of assignment scores, and the preference of English as the course language positively predicted the course performance. The latent variables of the perceived course achievement related to the observed variables of academic control and task value scale scores, and negatively predicted willingness for self-study after the course completion. In addition, the preference of English as the course language negatively predicted willingness for self-study in medical English ( Figure 3).

## Discussion

This study explored the attributive factors of students’ motivation to learn medical English via an online modality. We found that students' choice of English as the language of instruction and perceived course achievement were negative predictors of their motivation for English self-study after the class. Although we hypothesized a circulatory relationship among the variables of language for course delivery, performance in class, efficacy of English class, and willingness for further self-study, based on control-value theory, the observed results were contrary to the hypothesis.

Considering that choosing English as the course language was positively related to course performance, the reason for the negative relationship between the course language choice and willingness for self-study could be explained by self-satisfaction with course performance in highly performing students. These students might have been aware of their achievement in class based on their returned assignment grades, and concluded that it was unnecessary for them to undertake further study of medical English. Furthermore, the negative relationship between perceived course achievement (i.e., latent variable of the observed variables of academic control and task value scale scores) and the desire for future self-study may be interpreted as the impact of students’ self-satisfaction.

Another reason for the low motivation for self-study among those who chose English as the language of instruction might be that they want to complete their English learning in the classroom. They may feel that they are too busy to study on their own, and that self-study does not influence their grades. Presumably, those who chose English were not motivated to undertake self-study after the classes were conducted in Japanese.

Another possible reason for these results could be that the students preferred a collaborative face-to-face environment for learning medical English rather than individualized self-study. It is also important to note that the uncertainty and rapid changes caused by the COVID-19 pandemic have caused significant psychological distress among students, and medical students are exhausted by online learning ^18^.

The contradictory results of this study are explainable by Heckhausen’s action-phase model of developmental regulation. The theory suggests that individuals optimize their behaviors for goal engagement depending on the urgency of the goal to be achieved ^19^. For Japanese medical students, learning medical English may not be an urgent goal, and they prioritize the acquisition of medical knowledge and skills. The learning of medical English by medical students may be best facilitated by effective embedding within the long-term lifelong learning framework.

### Limitations

The results of this study were based on short-term investigations of medical students’ motivational briefs and course performance; however, it is essential to obtain longitudinal observations because language acquisition is a life-long learning process.

## Conclusion

We observed that Japanese medical students have complex values regarding learning medical English. The importance of incorporating the perspective of lifelong learning into the teaching of medical English was recognized.

## Data availability

### Underlying data

The raw data of this study are stored securely in the computer of the principal investigator of the article. The data cannot be made publicly available due to the security consideration reviewed by the IRB (ethical approval waived, decision date: January 8th, 2021). However, the raw data will be shared on reasonable request to the corresponding author (email: nomura_o@hirosaki-u.ac.jp) under conditions where the data provision is highly likely to develop medical education research and where the data can be secured in strict confidence.

### Extended data

Open Science Framework: Motivations of Japanese medical students to online medical English course. https://www.doi.org/10.17605/OSF.IO/PXD2U ^20^

This project contains the following extended data

- A copy of the pre-course survey
- A copy of the post-course survey

Data are available under the terms of the Creative Commons Zero "No rights reserved" data waiver (CC0 1.0 Public domain dedication).
