# Supplementary material for: Complex motivations of Japanese medical students to an online medical English course during the COVID-19 pandemic
Source: MedEdPublish (2016). 2022 Apr 6;12:25. [Version 1] doi: 10.12688/mep.19042.1 (PMC10111141; doi:10.12688/mep.19042.1)
Supplement: Peer review history

# Peer review: Reviewer response for version 1

**DOI:** 10.21956/mep.20400.r32567

**Published:** 2022-09-20

**Authors:** Yasushi Matsuyama

**Affiliations:** 1 Medical Education Center, Jichi Medical University, Shimotsuke, Japan

The data are appropriately validated and presented. I believe that this paper deserves to be indexed after minor revision.

 As for the minor revision, the authors should explain the details of the educational program based on educational theory in order for readers to generalize this research data and apply it to their education practice.

 #1:

 　In the introduction part, the authors explain the potential of online technology-based learning to enhance individual feedback and active teacher-student interactions in English language education in East Asia. I agree that individual feedback and active teacher-student interaction are key to student-centered and outcome-based education.

 　However, the statement "students can initiate learning modules at a convenient time and place, and watch videos of lectures by the instructors, which promotes student-centered and self-regulated learning" may mislead readers.　

 　Simply providing modules and on-demand videos for learning at any location and at any time is not student-centered education nor does it promote self-regulated learning. I think this part is a problem with the way the text is written, and I would appreciate it if the authors could revise it.

 #2:

 　In the various modalities of this online program, individualized feedback and active interaction between teachers and students online will motivate active learning. This could be the theory underlying the authors’ educational practice.

 　In other words, simply preparing on-demand materials, requesting students to submit a report, and providing one-time individual feedback on the submitted report is not an educational practice that takes advantages of online technology as explained in the introduction.

 　For this reason, in the educational context of the Methods section, I would like the authors to show how teachers and students were interactive through various modalities in the online learning course consisting of three modules. Specifically, I would like the authors to describe the following:

1. What methods did students use to communicate with teachers when they wanted to ask questions after watching the on-demand videos? And how often did they have communication with teachers?
2. What sort of learning tasks were given to students after they watched the video? Perhaps not just submitting a report, but what else did the students do?
3. When working on the assignment, can students ask questions to teachers in an online dialogue as needed?
4. How long did it take before students received feedback after submitting the assignment?
5. Did the authors try to improve the quality of teachers’ feedback in order to enhance students’ motivation?
6. After students received feedback, were they allowed to engage in continuous online interactions with faculty members?
7. Also, in terms of the impact of grading methods on student learning behavior, please indicate the level of stakes in the grading of the assignments submitted for this class.

#3:

 　In the Discussion or Limitations, I would also like to see how much the distinctive learning module itself, as indicated in #2, may have affected the motivation of the learners. Could there have been insufficient student/faculty interaction in some respects?

Have any limitations of the research been acknowledged?

Partly

Is the work clearly and accurately presented and does it cite the current literature?

Yes

If applicable, is the statistical analysis and its interpretation appropriate?

Yes

Are all the source data underlying the results available to ensure full reproducibility?

Yes

Is the study design appropriate and is the work technically sound?

Yes

Are the conclusions drawn adequately supported by the results?

Yes

Are sufficient details of methods and analysis provided to allow replication by others?

Partly

Reviewer Expertise:

Medical Education. Self-regulated learning. Assessment.

I confirm that I have read this submission and believe that I have an appropriate level of expertise to confirm that it is of an acceptable scientific standard, however I have significant reservations, as outlined above.

# Response

**Published:** 2023-03-01

**Authors:** Osamu Nomura

**Affiliations:** Hirosaki University, Japan

Thank you for the time and effort you have dedicated to providing insightful feedback, which has enabled us to strengthen our manuscript. We have revised the manuscript in response to the detailed suggestions and now wish to resubmit the paper for further consideration. We provide our revisions and changes in the point-by-point response below, and trust that these satisfactorily address all issues and concerns raised in your letter dated 20 Sep 2022.

 Comment #1: Thank you for indicating this point. Accordingly, we have included a more detailed explanation in the Introduction (page 4, para 3) as follows.  Students can initiate learning modules at a convenient time and place, watch videos of lectures recorded by the instructors, and ask questions online, which promotes student-centered and self-regulated learning.

 Comment #2: Response: Thank you very much for this important comment. In response, we have provided detailed explanations of each point in the Methods (page 4, Educational context) as follows. The first module consisted of reading an essay and submitting a reaction paper to it. Students were instructed to use skills taught in previous lectures and write 200–300 words. The second module consisted of writing an email to the person in charge of a desired study abroad destination. Three settings were presented to enable students to imagine and write in concrete terms. The third module was conducted in the second setting and comprised the following steps: (1) write a script for an elevator pitch, (2) practice, (3) record, (4) listen to the recording and perform a self-evaluation, and (5) complete a reflection sheet based on the self-evaluation. 

 The course materials were presented to the students to help them prepare for the next assignment. Because the submission requirements of the online course differed from the usual method, the students watched a video that explained how to submit their assignments smoothly (description of the e-learning platform and video submission).

 Communication with the students was by email, with the option of asking questions online; however, there were no instances of online conversations. Considering the loss of motivation over time, students who asked questions via email were responded to as soon as possible (within 24 hours at the latest).

 Feedback was provided within one week. Concise and specific feedback was provided promptly. Communication about feedback was by email, also with the option of asking questions online if necessary. All grading items for each assignment were clearly explained in each video and students’ submissions were graded accordingly.

 Comment #3:

 We consider that the extent to which the distinctive learning module itself affected the motivation of the learners has not yet been fully explored. We have acknowledged this point in the Discussion as follows (page 10, para 4). The online lecture and online submission of assignments allowed students to receive feedback immediately after submitting their assignments, which we believe led to a strong sense of interaction. We believe the interaction was facilitated by allowing students to watch the lecture and ask questions at a time of their choosing, whereas in a face-to-face class they would have to wait in line after class to do so, in the 10-minute window before their next class. We believe that this arrangement had a positive impact on their motivation in the second and subsequent lectures. However, due to the nature of the online course, the method of submitting assignments was unusual (description in the e-learning platform and video submission) and may have been stressful for the students. Again, thank you for giving us the opportunity to strengthen our manuscript in response to your valuable comments.

# Peer review: Reviewer response for version 1

**DOI:** 10.21956/mep.20400.r31857

**Published:** 2022-04-21

**Authors:** Maria Clelia Zurlo

**Affiliations:** 1 Dynamic Psychology Laboratory, Department of Political Sciences, University of Naples Federico II, Naples, Italy

The study aims to explore the relationship between Japanese medical students' motivations (including perceived academic control and task value) and assignment scores related to attending a course to learn medical English through an online modality.

 A structural equation model based on control value theory was used to examine the hypothesized relationship between the constructs. The study is interesting and useful. The methodology adopted for the data analysis is adequate.

 I have only two suggestions:

1. The purpose of the study must be better explained in the abstract.
2. In my opinion, the potential effects of the online modality of the course for learning medical English are not adequately explored. I suggest that the authors describe their potential influence on the results and/or introduce these aspects in the section containing limitations and future research.

Have any limitations of the research been acknowledged?

No

Is the work clearly and accurately presented and does it cite the current literature?

Yes

If applicable, is the statistical analysis and its interpretation appropriate?

Yes

Are all the source data underlying the results available to ensure full reproducibility?

Yes

Is the study design appropriate and is the work technically sound?

Yes

Are the conclusions drawn adequately supported by the results?

Yes

Are sufficient details of methods and analysis provided to allow replication by others?

Yes

Reviewer Expertise:

Perceived stress, motivations, psychological health conditions of university students

I confirm that I have read this submission and believe that I have an appropriate level of expertise to confirm that it is of an acceptable scientific standard.

# Response

**Published:** 2023-03-01

**Authors:** Osamu Nomura

**Affiliations:** Hirosaki University, Japan

Thank you for the time and effort you have dedicated to providing insightful feedback, which has enabled us to strengthen our manuscript. We have revised the manuscript in response to the detailed suggestions and now wish to resubmit the paper for further consideration. We provide our revisions and changes in the point-by-point response below, and trust that these satisfactorily address all issues and concerns raised in your letter dated 21 Apr 2022.

 Comment 1 Response: Thank you for pointing out this deficiency. In response, we have explained the purpose of the study in the Abstract in more detail (page 3, Background) as follows.

 Comment 2 Response: Thank you for mentioning this point. We have added the following to the Discussion section (page 10, para 4), as suggested.  The results suggest that characteristics of the online course, such as instructor grading (interaction) and the freedom to take the course and submit assignments at any time, increased students’ academic control and task value. The finding that students with high efficacy and those who chose English as their language of instruction did not want to further self-study with the course materials suggests that they tend to value the interaction of the online course, rather than their skill development. Again, thank you for giving us the opportunity to strengthen our manuscript in response to your valuable comments. We trust that the revisions are satisfactory and that the manuscript is now suitable for publication in your journal.
